# Psychometric Properties of the Dutch Version of the Dialectical Behavior Therapy Ways of Coping Checklist (DBT‐WCCL)

**DOI:** 10.1002/jclp.70077

**Published:** 2025-12-29

**Authors:** Carlijn J. M. Wibbelink, Roland Sinnaeve, Lindy‐Lou Boyette, Arnoud Arntz, Jan H. Kamphuis

**Affiliations:** ^1^ Department of Clinical Psychology University of Amsterdam Amsterdam the Netherlands; ^2^ Department of Neurosciences Mind Body Research, KU Leuven Leuven Belgium; ^3^ Current address: Clinical Psychology, University of Groningen Groningen the Netherlands; ^4^ Academic Center for Trauma and Personality Amsterdam the Netherlands; ^5^ Present address: Clinical Psychology University of Groningen Groningen The Netherlands

**Keywords:** borderline personality disorder, DBT skills, factor analysis, mechanisms of change, mediator, psychometrics

## Abstract

Dialectical behavior therapy (DBT) is an extensively studied treatment for borderline personality disorder (BPD), with skills use being one of the hypothesized mechanisms of change. Research has previously been hindered by the absence of an appropriate tool to measure skills use, leading to the development of the DBT Ways of Coping Checklist (DBT‐WCCL). The DBT‐WCCL aims to assess DBT skills use (DSS) as well as dysfunctional coping (DCS), which can be divided into dysfunctional coping in general (DCS1) and blaming others (DCS2). This study evaluated the Dutch version of the DBT‐WCCL by examining (1) the dimensional structure and measurement invariance across BPD and non‐clinical samples, (2) psychometric properties (reliability and validity), and (3) sensitivity to change. A total of 204 participants diagnosed with BPD and 103 non‐clinical controls completed the Dutch DBT‐WCCL along with instruments assessing emotion regulation and BPD manifestations. First, when including all DBT‐WCCL items, the hypothesized two‐factor and three‐factor models were not tenable due to substantial content overlap. When factor analyses included only items representing DBT skills or dysfunctional coping, support was found for three subscales (DSS, DCS1, and DCS2). Partial measurement invariance was established only for the DSS subscale. Reliability and known‐group validity were satisfactory for all scales, while inconclusive results were found for the concurrent validity of the DSS subscale. Finally, the DBT‐WCCL proved to be sensitive to change. In conclusion, our findings largely support the use of the Dutch DBT‐WCCL, but warrant caution when comparing samples on dysfunctional coping.

**Trial Registration:** The BOOTS study was registered in the Overview of Medical Research in the Netherlands (NL‐OMON21337).

## 1 Introduction

Borderline personality disorder (BPD) is one of the most common personality disorders with a prevalence rate of 1%−3% in the general population and 10%−22% in psychiatric populations (Ellison et al. 2018; Leichsenring et al. 2024). BPD is characterized by emotional instability, disturbed identity, impulsiveness, and interpersonal problems (American Psychiatric Association 2013). BPD is associated with low quality of life and a high level of suffering, marked by severe functional impairment, high comorbidity rates, and increased suicide risk and mortality rate (Dixon‐Gordon et al. 2015; Kjær et al. 2020; Laurenssen et al. 2016; Paris 2019; Tomko et al. 2014). Evidence‐based treatments for BPD are available, of which dialectical behavior therapy (DBT; Linehan 1993) is the most extensively studied treatment for BPD (Storebø et al. 2020). Numerous studies have demonstrated the effectiveness of DBT (for reviews, see, for instance, Hernandez‐Bustamante et al. 2024 and Kliem et al. 2010). Skills use has been proposed as a putative mechanism of change of DBT (Neacsiu, Rizvi, and Linehan 2010). To enable the investigation of skills use as a mechanism of change, reliable measurement of DBT skills use is essential, which is the focus of the present study.

DBT is a comprehensive treatment that combines elements from cognitive and behavioral treatments with acceptance and dialectical strategies (Linehan 1993). DBT includes four components: individual sessions, group skills training, between‐session consultation, and therapist team meetings. In DBT, the skills deficit model posits a central role for emotion regulation (Linehan 1993). More specifically, problematic behavior in individuals diagnosed with BPD is viewed as a consequence of dysregulated emotions or as dysfunctional attempts to regulate emotions (Neacsiu, Lungu, et al. 2014). As such, a significant emphasis in DBT is placed on teaching skills to help patients cope with emotion dysregulation and to use adaptive behavior instead of maladaptive behavior. These skills are grouped into four domains: mindfulness skills, interpersonal effectiveness skills, emotion regulation skills, and distress tolerance skills (Linehan 2015).

There is growing evidence for DBT skills use as a mechanism of change in DBT. After 1 year of DBT, skills use increased over time (Lindenboim et al. 2007; Neacsiu, Rizvi, Vitaliano, et al. 2010; Stepp et al. 2008). Moreover, several studies found that DBT skills use mediated improvements in treatment outcomes, including suicidality, non‐suicidal self‐injury, BPD symptoms, and emotional well‐being (Barnicot et al. 2016; Neacsiu, Rizvi, and Linehan 2010; O'Toole et al. 2012; Perroud et al. 2012). Although the number of studies examining DBT skills use is increasing, research has previously been hampered by the absence of an appropriate measurement tool (Neacsiu, Rizvi, Vitaliano, et al. 2010). Earlier research used a specific questionnaire to evaluate only one of the four DBT skills domains (e.g., O'Toole et al. 2012; Perroud et al. 2012), or diary cards were used to assess a range of DBT skills (e.g., Lindenboim et al. 2007; Stepp et al. 2008). However, diary cards include the names and acronyms of specific DBT skills, and they may therefore be more indicative of the patient's proficiency in DBT terminology than an accurate estimate of the use of skills (Neacsiu, Rizvi, Vitaliano, et al. 2010).

The absence of a reliable and valid measure to assess DBT skills use led to the development of the DBT Ways of Coping Checklist (DBT‐WCCL; Neacsiu, Rizvi, Vitaliano, et al. 2010). The DBT‐WCCL is a self‐report questionnaire derived from the Revised Ways of Coping Checklist (RWCCL; Vitaliano et al. 1985) with additional items to assess the full array of DBT skills. The DBT‐WCCL primarily aims to measure DBT skills use (DBT skills subscale [DSS]), with the remaining items functioning as distractors and measuring two types of dysfunctional coping (dysfunctional coping in general; Dysfunctional Coping Subscale 1 [DCS1], and blaming others; Dysfunctional Coping Subscale 2 [DCS2]). Neacsiu, Rizvi, Vitaliano, et al. (2010) recommended combining the two subscales measuring dysfunctional coping (i.e., DCS1 and DCS2) into one subscale (dysfunctional coping subscale [DCS]), while other studies (e.g., Burmeister et al. 2017; Hastings et al. 2022; Roder et al. 2014) have utilized these subscales separately. English, German, Italian, and Spanish versions of the DBT‐WCCL have been developed and validated (Arteaga‐de‐Luna et al. 2023; Burmeister et al. 2017; Neacsiu, Rizvi, Vitaliano, et al. 2010; Roder et al. 2014). The DBT‐WCCL, particularly the DSS, has demonstrated satisfactory reliability and validity across various populations, including patients diagnosed with BPD (Burmeister et al. 2017; Neacsiu, Rizvi, Vitaliano, et al. 2010), heterogeneous patient samples (Roder et al. 2014; Stein et al. 2016), and a non‐clinical sample (Arteaga‐de‐Luna et al. 2023). However, Burmeister et al. (2017) used a slightly adapted German version of the DBT‐WCCL with a different response format and item order, and Arteaga‐de‐Luna et al. (2023) proposed and evaluated a shortened version of the Italian DSS. A Dutch version of the DBT‐WCCL (Boyette et al. 2018) is currently used in a multicenter randomized clinical trial (RCT) into the effectiveness of DBT and schema therapy (ST) (the Borderline Optimal Treatment Selection [BOOTS] study; Wibbelink et al. 2022) to assess mechanisms of change in DBT. However, a psychometric evaluation of the Dutch version has not yet been conducted.

In the current study, the psychometric qualities of the Dutch adaptation of the DBT‐WCCL were studied in a non‐clinical sample and a BPD sample. First, we evaluated the factor structure as well as measurement invariance across non‐clinical controls and individuals with BPD. It was hypothesized that we would find support for a two‐factor model (Neacsiu, Rizvi, Vitaliano, et al. 2010) or three‐factor model (Burmeister et al. 2017), with one factor representing DBT skills and one or two factors representing dysfunctional coping skills. Measurement invariance across clinical and non‐clinical samples has not previously been evaluated and was therefore exploratively investigated in the present study. Second, we examined the reliability (internal consistency and item‐rest correlations) and validity (concurrent and known‐group validity) of the DBT‐WCCL in our samples. We expected adequate reliability and sufficient known‐group validity, as demonstrated by lower scores on the DSS subscale and higher scores on one or two subscales measuring dysfunctional coping skills in individuals with BPD prior to treatment, compared to non‐clinical controls. For concurrent validity, the DBT‐WCCL subscales were related to emotion dysregulation and the number and severity of BPD symptoms. The DBT model posits that emotion dysregulation and associated problematic behaviors partially stem from dysfunctional coping skills, and that the use of DBT strategies leads to improved emotion regulation (Boritz et al. 2018; Neacsiu, Eberle, et al. 2014). Therefore, it was hypothesized that the DSS subscale would have a negative correlation with both emotion dysregulation and the presence and severity of BPD manifestations. Conversely, the subscales measuring dysfunctional coping skills were expected to positively correlate with emotion dysregulation and the presence and severity of BPD manifestations. Finally, sensitivity to change was examined among BPD patients receiving DBT, whereby an increase in DBT skills use and a decrease in dysfunctional coping skills were expected.

## 2 Methods

### 2.1 Participants

The total sample included 204 individuals diagnosed with BPD, 39 of whom completed DBT, and 103 non‐clinical controls. Participants were 18 years or older and had sufficient proficiency in the Dutch language. BPD participants were included if they: (1) had BPD as their primary diagnosis, (2) had a BPD severity score above 20 on the Borderline Personality Disorder Severity Index, version 5 (BPDSI‐5), (3) were available and motivated for treatment and assessments, (4) did not meet the criteria in the past year for a psychotic disorder or bipolar I disorder with one or more manic episodes, (5) had not been diagnosed with antisocial personality disorder combined with violent interpersonal behavior in the past 2 years, (6) had an IQ above 80, (7) lived within a 45 min travel distance from the mental healthcare center, (8) had a permanent home address, and (9) had not received ST or DBT in the past year (Wibbelink et al. 2022). Non‐clinical controls were included if they: (1) did not report a diagnosis of a mental disorder or severe psychological complaints, (2) were not abusing alcohol or drugs, (3) had abstained from alcohol or drugs for at least 1 day, (4) had no brain injury, and (5) did not have an intellectual disability.

### 2.2 Procedure

Data of the BPD sample were drawn from an RCT on the effectiveness of DBT and ST (BOOTS study; Wibbelink et al. 2022) and collected between December 2019 and January 2024. Patients were recruited in several mental healthcare centers in the Netherlands. Screening of patients involved the assessment of disorders with the Structured Clinical Interview for Diagnostic and Statistical Manual of Mental Disorders 5th edition (DSM‐5) Personality Disorders (SCID‐5‐PD; First et al. 2015) and the Structured Clinical Interview for DSM‐5 Syndrome Disorders (SCID‐5‐S; First et al. 2018), as well as a screening interview and the BPDSI‐5. After a baseline assessment, patients were randomized to either DBT or ST. Data were collected through self‐report questionnaires and interviews at five assessment points: the pre‐treatment assessment (screening and baseline assessment) and four reassessments every 6 months until the end of the 2‐year treatment period. Only patients who completed DBT treatment and all five assessments at the time of data analysis were included in the sensitivity to change analysis. For detailed information on the procedure and DBT treatment protocol, the reader is referred to Wibbelink et al. (2022).

The non‐clinical controls were recruited using convenience sampling, via online websites (e.g., LinkedIn, Facebook) or face‐to‐face (family, friends, or relatives of researchers and students). The data collection of the control group took place in December 2022 and between December 2023 and January 2024 through online self‐report questionnaires.

### 2.3 Materials

#### 2.3.1 DBT‐WCCL

The DBT‐WCCL (Neacsiu, Rizvi, Vitaliano, et al. 2010) is an adaptation of the RWCCL (Vitaliano et al. 1985), with additional items to assess DBT skills use. The DBT‐WCCL includes 59 items measuring DBT skills (DSS; 38 items) and dysfunctional coping (DCS; 21 items), which may be divided into dysfunctional coping in general (DCS1; 15 items) and blaming others (DCS2; six items), depending on the results of the factor analysis. All items are rated on a four‐point Likert scale (0 = *never used* to 3 = *regularly used*) and focus on the past month. Mean scores were calculated for each scale. An overview of the items is provided in Appendix SA.

The English DBT‐WCCL was independently translated into Dutch by two translators, supported by suggestions from DBT experts. After this, another translator provided a back‐translation, which was then checked and approved by the original author of the DBT‐WCCL. The DBT‐WCCL has demonstrated adequate psychometric properties in other languages (Arteaga‐de‐Luna et al. 2023; Burmeister et al. 2017; Neacsiu, Rizvi, Vitaliano, et al. 2010; Roder et al. 2014).

#### 2.3.2 DERS‐SF

The Difficulties in Emotion Regulation Scale Short Form (DERS‐SF; Kaufman et al. 2016), a brief version of the DERS (Gratz and Roemer 2004), was used to assess emotion dysregulation. The DERS‐SF consists of 18 five‐point Likert scale items (1 = *almost never; 0%−10%* to 5 = *almost always; 91%−100%*), resulting in a total score ranging from 18 to 90. The DERS‐SF has shown satisfactory psychometric properties in several non‐Dutch languages (e.g., Danasasmita et al. 2024; Gouveia et al. 2022; Kaufman et al. 2016; Kim et al. 2024), consistent with the psychometric properties of the DERS, which has been validated in Dutch (Neumann et al. 2010).

#### 2.3.3 SCID‐5 BPD

The number of BPD symptoms was assessed in non‐clinical controls using the BPD section of the screening version of the SCID‐5‐PD (SCID‐5‐SPQ‐BPD; First et al. 2016), while the interview version was administered in the BPD sample (SCID‐5‐PD‐BPD; First et al. 2015). The SCID‐5‐SPQ‐BPD measures the nine DSM‐5 criteria with 15 dichotomous items (0 = *no*, 1 = *yes*). Each criterion is assessed by one item, except for criteria 3 (four questions), 5 (two questions), and 8 (three questions). Items belonging to the same criterion were combined into one item and assigned a score of 1 if at least one “yes” response was present. The total score ranges from 0 to 9. The SCID‐5‐PD‐BPD measures the nine DSM‐5 criteria with nine 3‐point Likert scale items (0 = *absent*, 1 = *subthreshold*, 2 = *present*), yielding a total score ranging from 0 to 18. Previous studies have found satisfactory psychometric properties for the SCID‐5‐SPQ as well as the SCID‐5‐PD (Bayad et al. 2021; Ekselius et al. 1994; Gharraee et al. 2021; Jacobsberg et al. 1995; Somma et al. 2017). The Dutch versions, however, have not yet been evaluated, although previous versions have been validated in Dutch (SCID‐II: Lobbestael et al. 2011; Weertman et al. 2003, SCID‐II‐PQ: Germans et al. 2012).

#### 2.3.4 BPDSI‐5

The BPDSI‐5 (Arntz et al. 2003; Giesen‐Bloo et al. 2010) was only administered in the BPD sample and assesses the severity of the nine DSM‐5 BPD criteria within the prior 3 months. The BPDSI‐5 is a semi‐structured interview comprising 70 items rated on an 11‐point Likert scale (0 = *never* to 10 = *daily*), except for the items measuring criterion 4 (Identity disturbance). These items are rated on a five‐point Likert scale (0 = *absent* to 4 = *dominant, clear, and well‐defined*) and then multiplied by 2.5. The total score is derived by summing the nine criteria scores and ranges from 0 to 90. The BPDSI‐5 is a modified version of the BPDSI‐IV (Arntz et al. 2003; Giesen‐Bloo et al. 2010), to which exact frequency scores were added, and some items were slightly rephrased. While the psychometric properties of the BPDSI‐5 have not been investigated, the Dutch BPDSI‐IV has been shown to be a valid and reliable measure (Giesen‐Bloo et al. 2010).

### 2.4 Data‐Analysis

First, the dimensional structure of the DBT‐WCCL was evaluated by testing two‐factor and three‐factor models including all items and both samples. Thereafter, separate factor models were examined including either the DSS items or DCS items, in line with previous research (Arteaga‐de‐Luna et al. 2023; Stein et al. 2016) and the way the questionnaire was developed and intended (Neacsiu, Rizvi, Vitaliano, et al. 2010). For the DSS items, a one‐factor model was tested, while for the DCS items one‐factor and two‐factor models were examined. Following recommendations by Rhemtulla et al. (2012), the factor models were analyzed using confirmatory factor analyses (CFA) with robust weighted least squares means and variances (WLSMV) and theta parameterization (Wells 2021), due to the Likert response format of the items. To evaluate model fit, the comparative fit index (CFI; Bentler 1990) and Tucker−Lewis Index (TLI; Tucker and Lewis 1973) were used, with values larger than 0.90 indicating an acceptable fit and values larger than 0.95 a good fit (Kline 2016). In addition, the root mean square error of approximation (RMSEA; Browne and Cudeck 1989) was applied, which should be lower than 0.08 or 0.06 to indicate an acceptable or good fit, respectively (Kline 2016). Modification indices were used to identify possible sources of model misspecification, including correlated measurement errors.

Second, measurement invariance of the factor models, including either the DSS or DCS items, was assessed across the two samples, using multiple‐group confirmatory factor analysis (MG‐CFA) with WLSMV as the estimation method and theta parameterization. Three levels of invariance were examined, including configural invariance, metric invariance, and scalar invariance. The fourth level, strict invariance, was excluded from the invariance tests, since testing residual invariance is not necessary to examine mean differences across groups (Putnick and Bornstein 2016). The comparative fit of the models was evaluated by examining the magnitude of change in the CFI, TLI, and RMSEA. A change larger than −0.010 for the CFI and TLI and larger than 0.015 for the RMSEA was considered a significant decrease in model fit (Chen 2007). Chi‐square statistics were not compared between the models, because this statistic tends to be overly sensitive with large samples (Putnick and Bornstein 2016). Modification indices were used as guidance to improve model fit by freeing parameters that strongly violated invariance.

Third, reliability (internal consistency and item‐rest correlations) and validity (concurrent and known‐group validity) were examined. Internal consistency was examined with Cronbach's *α* and McDonald's omega (*ω*), as well as their ordinal coefficients (Gadermann et al. 2012; Zumbo et al. 2007). *α* values larger than 0.80 and *ω* values larger than 0.75 were considered satisfactory (Watkins 2017). In addition, item‐rest correlations (*r*_ir_) were calculated, with values larger than 0.30 deemed satisfactory (Zijlmans et al. 2019). Next, concurrent validity was examined using Pearson's correlations. Known‐group validity was tested with independent‐samples *t*‐tests, combined with effect size coefficients (Cohen's *d*; 0.20 = small, 0.50 = medium, and ≥ 0.80 = large; Cohen 1992), as well as ANCOVAs including covariates to adjust for potential group differences in demographics. Finally, sensitivity to change was assessed among BPD patients who completed DBT and all assessments. Four paired‐samples *t*‐tests were conducted comparing pre‐treatment scores with scores on the reassessments. Additionally, effect sizes (Cohen's *d*) were calculated.

Two‐sided analyses were performed with *α* set at 0.05. Analyses were conducted using Mplus (CFA and MG‐CFA; version 8.8, Muthén and Muthén 1998−2017), R software (version 4.2.2; R Core Team 2022) with the psych package (internal consistency; Revelle 2024), and IBM SPSS (other statistical analyses; version 28.0.1.0; IBM Corp 2021).

## 3 Results

### 3.1 Descriptive Statistics

Demographic data of the BPD and non‐clinical control samples are shown in Table 1, including results of tests (independent‐samples *t‐*tests and chi‐square tests) for between‐group differences. Individuals with BPD differed significantly from the non‐clinical controls with respect to Dutch ethnicity and employment status (both lower in the patient group), whereas no significant differences were found for age, education level, and gender. Consequently, the between‐group analysis was performed twice, adjusting for Dutch ethnicity but not for employment status, which was considered a common contextual factor in individuals diagnosed with BPD (see Miller and Chapman 2001).

**Table 1:** Demographic data of the BPD group and non‐clinical control group.

	BPD		Non‐clinical		Analysis	
Characteristic	(*N* = 204)		(*N* = 103)			
	M	SD	M	SD	*t*	*p*
Age	32.21	9.57	33.02	13.90	0.53	0.594
Education^a^	4.19	1.65	4.41	1.54	1.12	0.263

	*N*	%	*N*	%	*χ* ^2^	*p*
Women	172	84.3	81	78.6	1.52	0.218
Dutch ethnicity	150	73.5	91	88.3	8.91	0.003
Employed	57	27.9	57	55.3	22.01	< 0.001

^a^ Based on the International Standard Classification of Education, 2011 version.

### 3.2 Factor Analyses

#### 3.2.1 CFA

Two‐factor and three‐factor models were fitted including all 59 items and all participants (*N* = 307). Results showed that the model fit of both the two‐factor model (*χ* ^2^[1651] = 3748.91, RMSEA = 0.064, 90% CI [0.062, 0.067], CFI = 0.773, TLI = 0.764) and three‐factor model (*χ* ^2^[1649] = 3590.52, RMSEA = 0.062, 90% CI [0.059, 0.065], CFI = 0.789, TLI = 0.782) was satisfactory according to the RMSEA, but unacceptable according to the CFI and TLI. Modification indices of both factor models suggested that model fit would be improved by freeing error correlations between a number of items as well as adding cross‐loadings between several DSS items and the DCS factor or DCS1/DCS2 factors. However, freeing up to 100 error correlations, adding cross‐loadings, and deleting items with a relatively low standardized factor loading ( ≤ 0.40) did not result in a satisfactory model fit for both factor models. Moreover, a post‐hoc exploratory factor analysis supported these findings, again yielding inconclusive results. While the three‐factor model showed near satisfactory fit indices, it also exhibited a substantial number of cross‐loadings (results are available upon request from the first author). Therefore, the analyses were continued with separate factor models including either the DSS items or the DCS items.

First, a one‐factor model was fitted including the 38 DSS items. The model demonstrated inadequate fit to the data, *χ* ^2^(665) = 1501.51, RMSEA = 0.064, 90% CI [0.060, 0.068], CFI = 0.877, TLI = 0.870. Modification indices were examined for the identification of error correlations that penalized model fit. A respecified model with four error correlations (items 19−23, 33−34, 34−57, and 42−57) resulted in an acceptable fit (*χ* ^2^[661] = 1294.15, RMSEA = 0.056, 90% CI [0.051, 0.060], CFI = 0.907, TLI = 0.901). Some item pairs reflected overlapping content (e.g., [19] “Focused on the good things in my life” and [23] “Focused on the good aspects of my life and gave less attention to negative thoughts or feelings”), whereas other items may be interpreted as an overrepresentation of a specific DBT subskill (i.e., comparing: [34] “Told myself things could be worse”, [42] “Thought how much better off I was than others”, and [57] “Compared myself to others who are less fortunate”). Additionally, standardized factor loadings are reported in Appendix SB (Table B1), with one factor loading below 0.30 (item 29).

Second, for the DCS items, one‐factor and two‐factor models were evaluated. The one‐factor model fitted poorly to the data (*χ* ^2^[189] = 875.19, RMSEA = 0.109, 90% CI [0.102, 0.116], CFI = 0.868, TLI = 0.853). However, the fit of the two‐factor model was satisfactory (*χ* ^2^[188] = 486.42, RMSEA = 0.072, 90% CI [0.064, 0.080], CFI = 0.943, TLI = 0.936), indicating a bidimensional factor structure of the DCS items. The latent correlation between the two factors was large (*r* = 0.61). None of the standardized factor loadings were below 0.30 (see Appendix SB, Table B1).

#### 3.2.2 Measurement Invariance

Measurement invariance across individuals with BPD and non‐clinical controls was assessed separately for the one‐factor model including the DSS items and the two‐factor model including the DCS items. The results of the measurement invariance tests are presented in Table 2. First, measurement invariance was assessed for the DSS items using the unidimensional factor model with four error correlations. The one‐factor model with the DSS items showed configural invariance with acceptable model fit. Next, the metric invariance model yielded a slightly better model fit than the configural invariance model, indicating that the factor loadings were equal across groups. Subsequently, thresholds were constrained across groups to assess scalar invariance. The scalar invariance model showed a substantial decrease in fit compared to the metric invariance model according to the CFI and TLI. Using modification indices, threshold constraints of four items were sequentially identified and freed before a partial scalar invariance model was identified that did not significantly differ in model fit from the metric invariance model. The standardized estimates of the non‐invariant thresholds are shown in Appendix SB, Table B2.

**Table 2:** Results of measurement invariance tests.

Model	*χ* ^2^ (df)	RMSEA (90% CI)	CFI	TLI	ΔRMSEA	ΔCFI	ΔTLI
DSS items							
Configural	1890.08 (1322)	0.053 (0.047; 0.058)	0.909	0.903			
Metric	1893.45 (1359)	0.051 (0.045; 0.056)	0.914	0.911	−0.002	0.005	0.008
Scalar	2103.17 (1434)	0.055 (0.050; 0.060)	0.893	0.895	0.004	−0.021	−0.016
Scalar^a^	2013.23 (1426)	0.052 (0.046; 0.057)	0.906	0.907	0.001	−0.008	−0.004
DCS items							
Configural	712.89 (376)	0.076 (0.068; 0.085)	0.890	0.877			
Configural^b^	641.53 (372)	0.069 (0.060; 0.078)	0.912	0.900	−0.007	0.022	0.023
Metric^b^	615.57 (391)	0.061 (0.052; 0.070)	0.926	0.921	−0.008	0.014	0.021
Scalar^b^	758.38 (431)	0.070 (0.062; 0.079)	0.893	0.895	0.009	−0.033	−0.026
Scalar^b^, ^c^	672.22 (415)	0.064 (0.055; 0.072)	0.916	0.915	0.003	−0.010	−0.006

Abbreviations: DCS = dysfunctional coping subscale, DSS = DBT skills subscale.

^a^ Thresholds were freed for items 6, 19, 29, and 42. This model was compared with the metric model.

^b^ Including error correlations between items 3 and 14 and items 17 and 20.

^c^ Thresholds were freed for items 3, 7, 12, 17, 20, 37, 41, and 48. This model was compared with the metric model.

Second, measurement invariance was tested for the DCS items using the bidimensional factor model. However, non‐clinical controls did not score a “3” on item 30, resulting in an unequal number of categories on item 30 between individuals with BPD and non‐clinical controls. Combining categories or treating the items as continuous by using Maximum Likelihood instead of WLSMV as the estimation method were not considered appropriate strategies. Therefore, a score of “2” on item 30 of a randomly selected non‐clinical participant was changed to a score of “3”, and the analyses proceeded using the modified data set. This process was repeated 10 times to rule out chance findings and yielded similar outcomes. Moreover, CFA with the two‐factor model using the modified data sets resulted in nearly identical results.

For configural invariance, model fit was considered acceptable based on the RMSEA, but unsatisfactory according to the CFI and TLI. The modification indices indicated error correlations between items 3 and 14 and items 17 and 20, which contained similar content (e.g., “Blamed myself” and “Criticized or lectured myself”). Including these error correlations improved the model fit to a satisfactory level. Subsequently, factor loadings were constrained across groups which yielded a better model fit compared to the configural model. Finally, constraining item thresholds to assess scalar invariance resulted in a significant decrease in model fit based on the CFI and TLI. Using modification indices, threshold constraints of eight items were sequentially identified and released, resulting in a partial scalar invariance model that was not significantly different in model fit from the metric invariance model (see Appendix SB, Table B3 for the standardized estimates of the thresholds of the non‐invariant items). Following recommendations in the literature that allow releasing a maximum of 20% of parameter constraints to consider models as partially invariant (Bowen and Masa 2015; Dimitrov 2010), we concluded that scalar invariance was not tenable.

### 3.3 Reliability and Validity

Results of the reliability and validity analyses are shown in Table 3. First, the internal consistencies of the three scales proved to be good in the total group as well as in the specific groups. Item‐rest correlations were mostly larger than 0.30, except for items 13 (total group and BPD group), 29 (total group and non‐clinical group), 31 (BPD group), and 35 (BPD group) of the DSS scale and item 37 (BPD group) of the DCS1 scale (see Appendix SB, Tables B4−B6). However, removing these items did not lead to substantial improvements in the internal consistencies that would justify their removal. Second, significant negative correlations in the total and BPD group were found between DSS and emotion dysregulation, suggesting that in these groups, greater DBT skills use was related to less emotion dysregulation. However, in contrast to the hypothesis, no significant associations were found between DSS and the presence or severity of BPD symptoms, except for a weak negative correlation in the total group. In addition, the associations between emotion dysregulation, presence and severity of BPD symptoms, and both DCS1 and DCS2 were significant, except for the associations between DCS2 and presence of BPD symptoms or emotion dysregulation in the non‐clinical group. This suggests that, in general, more use of dysfunctional coping, including general dysfunctional coping and blaming others, was related to a higher presence and severity of BPD symptoms and more emotion dysregulation. Finally, individuals with BPD scored significantly lower on DBT skills use (DSS) and higher on general dysfunctional coping (DCS1) and blaming others (DCS2) compared to non‐clinical controls, with small‐to‐medium (DSS), medium (DCS2), and large (DCS1) effect sizes. However, the between‐group differences on the DCS scales should be interpreted with caution, as scalar invariance did not hold.

**Table 3:** Reliability and validity of the DBT‐WCCL in the total group, BPD group, and non‐clinical control group.

Psychometric property	DSS	DCS1	DCS2
Internal consistency (*α*/ordinal *α*)			
Total group	0.92/0.94	0.90/0.92	0.81/0.85
BPD group	0.92/0.93	0.82/0.86	0.80/0.84
Non‐clinical	0.94/0.95	0.87/0.88	0.78/0.81
Internal consistency (*ω*/ordinal *ω*)			
Total group	0.93/0.95	0.92/0.94	0.88/0.90
BPD group	0.92/0.94	0.85/0.89	0.87/0.90
Non‐clinical group	0.95/0.95	0.89/0.90	0.83/0.88
Mean item‐rest correlations (*r*_ir_)			
Total group	0.48	0.58	0.56
BPD group	0.45	0.44	0.56
Non‐clinical group	0.53	0.52	0.52
Concurrent validity (*r*)			
Emotion dysregulation (DERS‐SF)			
Total group	−0.23***	0.75***	0.35**
BPD group	−0.20**	0.57***	0.30***
Non‐clinical group	−0.06	0.47***	0.12
Number of BPD symptoms (SCID‐5‐PD/SPQ)			
Total group^a^ ^,^ ^b^	−0.12*	0.60***	0.33***
BPD group	0.07	0.15*	0.25**
Non‐clinical group^b^	0.05	0.28**	0.11
Severity of BPD symptoms (BPDSI‐5)^c^	0.07	0.33**	0.29**
Known‐group validity			
Mean BPD group (SD)	1.53 (0.46)	2.13 (0.47)	1.25 (0.70)
Mean non‐clinical group (SD)	1.70 (0.50)	1.28 (0.54)	0.90 (0.54)
* t*‐value/*F*‐value^d^	3.08**/9.58**	−14.24***/198.82***	−4.93***/18.20***
Cohen's *d*/corrected Cohen's *d* d	0.37/0.38	−1.72/−1.73	−0.55/−0.52

Abbreviations: BPDSI‐5 = borderline personality disorder severity index version 5, DCS1 = dysfunctional coping subscale 1 ‐ general dysfunctional coping, DCS2 = dysfunctional coping subscale 2 ‐ blaming others, DSS = DBT skills subscale, SCID‐5‐PD = structured clinical interview for DSM‐5 personality disorders, SCID‐5‐SPQ = structured clinical interview for DSM‐5 Screening Personality Questionnaire.

^a^ Since the range of the SCID‐5‐PD scores was twice as large as the range of the SCID‐5‐SPQ scores, the SCID‐5‐PD scores were first divided by two before being combined into one variable.

^b^ Spearman correlations were calculated due to a severe violation of the normality assumption.

^c^ The BPDSI‐5 was only administered in the BPD group.

^d^ Based on ANCOVA, corrected for Dutch ethnicity.

* *p* < 0.05

** *p* < 0.01

*** *p* < 0.001.

### 3.4 Sensitivity to Change

The sensitivity to change analysis included 39 individuals with BPD who completed 2 years of DBT as well as the assessments. The DBT‐WCCL was administered before treatment and every 6 months during treatment. Table 4 presents the mean scores at each assessment and the results of the four paired‐samples *t*‐tests. First, significant improvements were observed in DBT skills use between the pre‐treatment assessment and reassessments, with moderate‐to‐large to large effect sizes. Notably, the difference between the pre‐treatment assessment and the end‐of‐treatment assessment (24 months) was smaller than the differences between the pre‐treatment assessment and earlier reassessments. Moreover, an exploratory analysis showed a significant decrease in DBT skills use between 18 and 24 months of treatment, *t*(38) = ‐2.65, *p* = 0.012, *d* = −0.42. Second, significant decreases with large effect sizes were found in general dysfunctional coping (DCS1). Finally, significant decreases in blaming others (DCS2) were found between the pre‐treatment assessment and reassessments at 12, 18, and 24 months of treatment, with moderate to moderate‐to‐large effect sizes. However, no significant decrease was found between the pre‐treatment assessment and reassessment after 6 months of treatment.

**Table 4:** Descriptives and paired samples *t*‐test statistics for the assessments before and during treatment.

	DSS				DCS1				DCS2			
Assessment	M	∆^a^	*t*‐value	Cohen's *d*	M	∆^a^	*t*‐value	Cohen's *d*	M	∆^a^	*t*‐value	Cohen's *d*
Pre‐treatment	1.55				2.11				1.18			
6 months	1.96	0.41	7.19***	1.15	1.75	−0.36	−5.16***	−0.83	1.04	−0.14	−1.50	−0.24
12 months	2.02	0.48	7.84***	1.26	1.36	−0.75	−6.92***	−1.11	0.82	−0.35	−3.07**	−0.49
18 months	1.99	0.45	6.83***	1.09	1.31	−0.80	−7.34***	−1.18	0.71	−0.47	−4.26***	−0.68
24 months	1.86	0.32	4.21***	0.67	1.30	−0.82	−7.50***	−1.20	0.74	−0.44	−4.46***	−0.71

Abbreviations: DCS1 = dysfunctional coping subscale 1 ‐ general dysfunctional coping, DCS2 = dysfunctional coping subscale 2 ‐ blaming others, DSS = DBT skills subscale.

^a^ Reassessment score minus pre‐treatment score.

** *p* < 0.01

*** *p* < 0.001.

## 4 Discussion

We conducted an in‐depth psychometric evaluation of the Dutch adaptation of the DBT‐WCCL that included the assessment of (1) the dimensional structure and measurement invariance across BPD and non‐clinical samples, (2) psychometric properties (reliability and validity), and (3) sensitivity to change. First, when including all DBT‐WCCL items, the two‐factor and three‐factor models—comprising one factor (DSS) representing DBT skills use and one (DCS) or two factors (DCS1/DCS2) representing dysfunctional coping—were not tenable. Several items showed substantial cross‐loadings, likely due to content overlap between the scales, which stemmed from the development process of the DBT‐WCCL. However, including either the DSS items or DCS items to examine separate factor models yielded the expected dimensional structure. A unidimensional factor model for the DSS items was found, which demonstrated measurement invariance. A bidimensional factor model representing general dysfunctional coping (DCS1) and blaming others (DCS2) was identified for the DCS items, but scalar invariance could not be established. Second, reliability and known‐group validity were satisfactory for all three scales, while inconclusive results were found for concurrent validity. Finally, the DBT‐WCCL proved to be sensitive to change.

The hypothesized two‐factor or three‐factor structure for the DBT‐WCCL items (Neacsiu, Rizvi, Vitaliano, et al. 2010) was not replicated in the Dutch version of the DBT‐WCCL. This is partly in line with the German version of the DBT‐WCCL, of which the initial fit was unacceptable, and a satisfactory fit was achieved only after the removal of several items (Burmeister et al. 2017). However, the German version uses a different response format (5‐point Likert scale) and a different order of items. The inadequate fit, partly due to the cross‐loadings between the DBT skills use items and the one or two factors representing dysfunctional coping, is not surprising considering the development process of the DBT‐WCCL. The RWCCL (Vitaliano et al. 1985) was used as a base measure for the DBT‐WCCL with the addition of items reflecting DBT skills (Neacsiu, Rizvi, Vitaliano, et al. 2010). Twenty‐two RWCCL items were classified as DBT skills, while 21 RWCCL items were classified as not being a DBT skill and included in the questionnaire as distractors. Therefore, the DBT‐WCCL was not specifically developed to measure multiple distinct constructs; its primary aim is to measure DBT skills use, with the remaining items turning out to measure dysfunctional coping (Neacsiu, Rizvi, Vitaliano, et al. 2010). Furthermore, considering that more than half of the DBT skills use items were originally derived from the RWCCL, it is not surprising that substantial cross‐loadings emerged and, as a result, the multifactor models including all items were not tenable. In addition, some items can be interpreted as both functional behavior, representing a skill, or as dysfunctional behavior (Burmeister et al. 2017). For example, item 6 (“Made sure I'm responding in a way that doesn't alienate others”) may reflect functional interpersonal behavior, but it could also indicate dysfunctional coping driven by a fear to be rejected by others (Burmeister et al. 2017). Likewise, item 13 (“Treated myself to something really tasty”) can be interpreted as a DBT skill, while it can also be related to dysfunctional eating behavior, which is related to BPD (Marino and Zanarini 2001).

Therefore, in line with previous research (Arteaga‐de‐Luna et al. 2023; Stein et al. 2016), separate factor models were examined including either the DSS items or the DCS items, which demonstrated the expected dimensional structure. All DSS items loaded onto one factor representing DBT skills use. As in the Spanish DBT‐WCCL (Arteaga‐de‐Luna et al. 2023), item 29 (“Soothed myself by surrounding myself with a nice fragrance of some kind”) exhibited a low factor loading. This might be related to it having the lowest endorsement frequency of all DBT skills (DeVellis 2016). In addition, it was necessary to model the covariance between items with similar content, consistent with Stein et al. (2016). This may also indicate a bloated specific factor (Cattell and Tsujioka 1964), reflecting an overrepresentation of a narrow construct (Watts et al. 2023). Specifically, this concerns the subskill “Comparing”, which is part of the distress tolerance skills grouped under the acronym ACCEPTS. For future use of the DSS subscale, it may be advisable to eliminate redundant items, as has already been done in the Spanish version of the DBT‐WCCL (Arteaga‐de‐Luna et al. 2023). With respect to the DCS items, the two‐factor solution provided a better fit than the one‐factor solution, indicating that these items capture general dysfunctional coping and blaming others as distinct but related constructs.

Next, measurement invariance across individuals with BPD and non‐clinical controls was examined for the two separate factor models. For the factor model with DSS items, measurement invariance was demonstrated after releasing the thresholds of four items (items 6, 19, 29, and 42). The thresholds of items 6 and 29 were larger in the non‐clinical control sample compared to the BPD sample, whereas the thresholds of items 19 and 42 were larger in the BPD sample than in the non‐clinical control sample. This suggests that non‐clinical controls scored lower on item 6 (“Made sure I'm responding in a way that doesn't alienate others”) and item 29 (“Soothed myself by surrounding myself with a nice fragrance of some kind”) and individuals with BPD scored lower on item 19 (“Focused on the good things in my life”) and item 42 (“Thought how much better off I was than others”) than would be expected based on their position on the underlying factor (DBT skills use). As fewer than 20% of the thresholds were released, we concluded that partial scalar invariance was established (Bowen and Masa 2015; Dimitrov 2010). However, scalar invariance was not tenable for the factor model with DCS items, as it required the release of 38% of the thresholds. This suggests that comparisons between BPD and non‐clinical groups are possible for DBT skills use, but not for general dysfunctional coping and blaming others (Steinmetz 2013).

In accordance with previous research (Arteaga‐de‐Luna et al. 2023; Burmeister et al. 2017; Neacsiu, Rizvi, Vitaliano, et al. 2010; Roder et al. 2014; Stein et al. 2016), the DBT‐WCCL proved to be a reliable questionnaire in our Dutch samples. In addition, known‐group validity was satisfactory for all three scales; however, inconclusive results were found for concurrent validity. For the DCS subscales, concurrent validity was largely supported. Contrary to the hypothesis, the concurrent validity of the DSS subscale was questionable. Emotion dysregulation was related to lower DBT skills use, except within the non‐clinical sample. However, in general, no significant associations were found between DBT skills use and the presence or severity of BPD manifestations. This finding contrasts previous research by Roder et al. (2014) and Stepp et al. (2008), but aligns with the study of Southward et al. (2023). The lack of a significant association is possibly related to the way skills use is assessed in the DBT‐WCCL. The DBT‐WCCL assesses the frequency of skills use, but it does not provide information on the quality or effectiveness of its use (Neacsiu, Rizvi, Vitaliano, et al. 2010). Emerging evidence suggests that frequency of skills use is less important compared to the quality, effectiveness, and timing of these skills (Southward and Cheavens 2020; Southward et al. 2023). In the present study, the DBT‐WCCL was assessed among individuals without knowledge of DBT, meaning they had not been trained in when and how to use certain skills. It is possible that some individuals with higher BPD severity may have used more skills but in a less efficient manner. In addition, several DSS items can be interpreted as both functional and dysfunctional behavior (Burmeister et al. 2017), which may have obscured the association between DBT skills use and BPD manifestations. Alternatively, although previous research suggests a relationship between DBT skills use and BPD severity (Barnicot et al. 2016; Perroud et al. 2012; Roder et al. 2014; Stepp et al. 2008), BPD is a complex disorder and this relationship may be more nuanced, for example, depending on the specific skills domain, influenced by moderators, or (partially) mediated by other factors (Neacsiu, Rizvi, Vitaliano, et al. 2010; Stepp et al. 2008; Southward et al. 2023). To conclude, more research into the relation between DBT skills use and BPD is necessary.

Finally, sensitivity to change was demonstrated for the DBT‐WCCL. In a sample of BPD patients receiving DBT, use of DBT skills increased, and scores on dysfunctional coping (general dysfunctional coping and blaming others) decreased substantially between the pre‐treatment assessment and reassessments during treatment. Notably, DBT skills use decreased in the last half year of treatment, between 18 and 24 months of treatment. Other studies on the sensitivity of change of the DBT‐WCCL focused on treatment durations ranging from 8 days to 4 months (Burmeister et al. 2017; Neacsiu, Rizvi, Vitaliano, et al. 2010; Stein et al. 2016), limiting direct comparisons. However, previous research found that the most significant learning of DBT skills occurred in the first months of treatment, with less or no increases in the last months of treatment (Barnicot et al. 2016; McMain et al. 2022; Neacsiu, Rizvi, and Linehan 2010; Robinson et al. 2018; Southward et al. 2023). In addition, treatment intensity of DBT in our study decreased drastically after 12 months of treatment, potentially limiting opportunities for learning and practicing DBT skills, which may have resulted in less DBT skills use. Finally, the decrease in DBT skills use at the end of treatment could also be related to the distribution of DBT‐WCCL items across the four skills domains, as half of the items were classified as distress tolerance skills (Neacsiu, Rizvi, Vitaliano, et al. 2010). Stepp et al. (2008) suggested that the use of distress tolerance skills might decline at the end of treatment, indicating increased behavioral control and problem‐solving abilities. The instrument might therefore benefit from a critical review on the distribution of items measuring the four skills domains, as a more balanced representation may enhance the sensitivity to change.

This study has several limitations. First, the measurement invariance of the factor model including DCS items was examined with modified data sets due to an unequal number of categories for item 30. A modified data set was created by changing a “2”‐score on item 30 to a “3”‐score of a randomly selected non‐clinical participant, and this process was repeated 10 times. Although these modified data sets yielded similar outcomes and the CFA using the modified data sets produced nearly identical results to the CFA with the original data set, this analytic strategy was considered suboptimal. More research into the measurement invariance of the DBT‐WCCL, specifically the DCS subscales, is therefore necessary. Moreover, measurement invariance across individuals diagnosed with BPD and other clinical samples remains to be investigated. Second, the concurrent validity of the DSS subscale was ideally evaluated by examining the association with another instrument measuring DBT skills. However, this was not possible due to an absence of a reliable DBT skills measure (Burmeister et al. 2017; Neacsiu, Rizvi, Vitaliano, et al. 2010). As a result, definitive conclusions regarding the concurrent validity of the DSS subscale cannot be drawn. In addition, the instruments used to assess concurrent validity (DERS‐SF, BPDSI‐5, SCID‐5‐PD, and SCID‐5‐SPQ) have not yet been validated in Dutch, meaning that the results regarding concurrent validity should be interpreted with some caution. Nevertheless, previous or extended versions of these instruments have been evaluated in the Dutch language. Moreover, we did not include measures to assess PD severity or maladaptive traits as defined in the DSM‐5 Alternative Model for Personality Disorders (American Psychiatric Association 2013) or International Statistical Classification of Diseases and Related Health Problems (11th ed.; ICD‐11; World Health Organization 2019). Future research should incorporate these measures for a more comprehensive evaluation. Third, the BPD sample differed from the non‐clinical sample on employment status and Dutch ethnicity. The between‐group analyses were therefore conducted twice, correcting for Dutch ethnicity. However, correcting for employment status was not recommended (Miller and Chapman 2001), as the difference in employment status was considered related to BPD (Sansone and Wiederman 2013). Finally, the data of the BPD sample were partly collected during the COVID‐19 pandemic, which may have affected the results. Previous research has reported conflicting results regarding the impact of the pandemic on individuals with BPD, varying from a reduction (Salamin et al. 2021) to an increase (Heidari et al. 2022; McLoughlin et al. 2022; Schulze et al. 2022) in BPD manifestations. Moreover, the lockdowns created fewer opportunities for social interaction, which may have decreased the need for individuals with BPD to use interpersonal skills, potentially influencing the results related to DBT skills use.

In sum, for the Dutch version of the DBT‐WCCL, the subscales exhibited substantial content overlap, leading to the inability to establish the hypothesized two‐factor or three‐factor structure when including all DBT‐WCCL items. However, when factor analyses were conducted including only items representing DBT skills or dysfunctional coping—more in line with the way the questionnaire was developed and intended—support was found for the existence of the DBT skills use subscale (DSS), as well as two subscales measuring dysfunctional coping (general dysfunctional coping, DCS1, and blaming others, DCS2). Partial measurement invariance was established for the DSS subscale, allowing for comparisons between individuals with BPD and non‐clinical samples with respect to DBT skills use. Moreover, adequate reliability and known‐group validity were found for the DBT‐WCCL subscales, while inconclusive results were found for concurrent validity. Finally, the satisfactory sensitivity to change of the DBT‐WCCL subscales enables the utilization of the DBT‐WCCL to measure DBT skills use and dysfunctional coping over time. To conclude, our preliminary evidence largely supports the use of the Dutch version of the DBT‐WCCL for tracking DBT skills use, but we recommend caution in using the DCS subscales, particularly when making comparisons between clinical and non‐clinical samples. Moreover, more research is needed, for example to examine measurement invariance and concurrent validity more extensively, and there is still room for improvement, including reducing content overlap between scales and achieving a more balanced distribution of items across the various DBT skills domains. As research on DBT skills use has previously been hindered by the lack of an appropriate measurement tool (Neacsiu, Rizvi, Vitaliano, et al. 2010), the development of the DBT‐WCCL, along with its availability in various languages, makes a valuable contribution to research (on mechanisms of change) in DBT.

## Funding

The funding bodies had no role in the design, collection, analysis, and interpretation of the data. The study including the non‐clinical participants did not receive funding.

## Ethics Statement

The BOOTS study received approval from the Medical Ethics Committee of the Academic Medical Center Amsterdam (registration number NL66731.018.18). The study including the non‐clinical participants was approved by the Ethics Review Board of the Faculty of Social and Behavioural Sciences, University of Amsterdam (registration number FMG‐5576_2023). We obtained signed informed consents from all participants in the study.

## Conflicts of Interest

The authors declare no conflicts of interest.

## Supporting information

Appendix A.

Appendix B.

## Data Availability

As the participant‐level data set could contain information that compromises the anonymity of the clinical participants, this will not be made publicly available. The data that support the findings of this study are available from the corresponding author (C.J.M.W.) upon reasonable request.
