# Supplementary material for: Psychometric Properties of the Dutch Version of the Dialectical Behavior Therapy Ways of Coping Checklist (DBT‐WCCL)
Source: J Clin Psychol. 2025 Dec 29;82(3):338–49. doi: 10.1002/jclp.70077 (PMC12882799; doi:10.1002/jclp.70077)
Supplement: Supplementary file 1 — Appendix A. [file JCLP-82-338-s002.docx]

**Appendix A**

**Overview of DBT-WCCL Items (Neacsiu, Rizvi, Vitaliano et al., 2010)**

1. Bargained or compromised to get something positive from the situation.

2. Counted my blessings.

3. Blamed myself.

4. Concentrated on something good that could come out of the whole thing.

5. Kept feelings to myself.

6. Made sure I'm responding in a way that doesn’t alienate others.

7. Figured out who to blame.

8. Hoped a miracle would happen.

9. Tried to get centered before taking any action.

10. Talked to someone about how I’ve been feeling.

11. Stood my ground and fought for what I wanted.

12. Refused to believe that it had happened.

13. Treated myself to something really tasty.

14. Criticized or lectured myself.

15. Took it out on others.

16. Came up with a couple of different solutions to my problem.

17. Wished I were a stronger person — more optimistic and forceful.

18. Accepted my strong feelings, but not let them interfere with other things too much.

19. Focused on the good things in my life.

20. Wished that I could change the way that I felt.

21. Found something beautiful to look at to make me feel better.

22. Changed something about myself so that I could deal with the situation better.

23. Focused on the good aspects of my life and gave less attention to negative thoughts or feelings.

24. Got mad at the people or things that caused the problem.

25. Felt bad that I couldn't avoid the problem.

26. Tried to distract myself by getting active.

27. Been aware of what has to be done, so I've been doubling my efforts and trying harder to make things work.

28. Thought that others were unfair to me.

29. Soothed myself by surrounding myself with a nice fragrance of some kind.

30. Blamed others.

31. Listened to or played music that I found relaxing.

32. Gone on as if nothing had happened.

33. Accepted the next best thing to what I wanted.

34. Told myself things could be worse.

35. Occupied my mind with something else.

36. Talked to someone who could do something concrete about the problem.

37. Tried to make myself feel better by eating, drinking, smoking, taking medications, etc.

38. Tried not to act too hastily or follow my own hunch.

39. Changed something so things would turn out right.

40. Pampered myself with something that felt good to the touch (e.g., a bubble bath or a hug).

41. Avoided people.

42. Thought how much better off I was than others.

43. Just took things one step at a time.

44. Did something to feel a totally different emotion (like gone to a funny movie).

45. Wished the situation would go away or somehow be finished.

46. Kept others from knowing how bad things were.

47. Focused my energy on helping others.

48. Found out what other person was responsible.

49. Made sure to take care of my body and stay healthy so that I was less emotionally sensitive.

50. Told myself how much I had already accomplished.

51. Made sure I respond in a way so that I could still respect myself afterwards.

52. Wished that I could change what had happened.

53. Made a plan of action and followed it.

54. Talked to someone to find out about the situation.

55. Avoided my problem.

56. Stepped back and tried to see things as they really are.

57. Compared myself to others who are less fortunate.

58. Increased the number of pleasant things in my life so that I had a more positive outlook.

59. Tried not to burn my bridges behind me, but leave things open somewhat.
