# Supplementary material for: Psychometric Properties of the Dutch Version of the Dialectical Behavior Therapy Ways of Coping Checklist (DBT‐WCCL)
Source: J Clin Psychol. 2025 Dec 29;82(3):338–49. doi: 10.1002/jclp.70077 (PMC12882799; doi:10.1002/jclp.70077)
Supplement: Supplementary file 2 — Appendix B. [file JCLP-82-338-s001.docx]

**Appendix B**

**Factor Loadings, Thresholds, Item-Rest Correlations, and Mean Scores of the DBT-WCCL Items**

Table B1

*Standardized Factor Loadings Based on the One-Factor Model of DSS Items and Two-Factor Model of DCS Items*

| Item | DSS | DCS1 | DCS2 |
| --- | --- | --- | --- |
| 1 | .48 |  |  |
| 2 | .59 |  |  |
| 3 |  | .70 |  |
| 4 | .66 |  |  |
| 5 |  | .46 |  |
| 6 | .37 |  |  |
| 7 |  |  | .71 |
| 8 |  | .58 |  |
| 9 | .60 |  |  |
| 10 | .49 |  |  |
| 11 | .47 |  |  |
| 12 |  | .56 |  |
| 13 | .31 |  |  |
| 14 |  | .61 |  |
| 15 |  |  | .65 |
| 16 | .66 |  |  |
| 17 |  | .83 |  |
| 18 | .61 |  |  |
| 19 | .71 |  |  |
| 20 |  | .84 |  |
| 21 | .51 |  |  |
| 22 | .53 |  |  |
| 23 | .66 |  |  |
| 24 |  |  | .70 |
| 25 |  | .81 |  |
| 26 | .48 |  |  |
| 27 | .58 |  |  |
| 28 |  |  | .80 |
| 29 | .29 |  |  |
| 30 |  |  | .79 |
| 31 | .38 |  |  |
| 32 |  | .49 |  |
| 33 | .47 |  |  |
| 34 | .48 |  |  |
| 35 | .34 |  |  |
| 36 | .56 |  |  |
| 37 |  | .64 |  |
| 38 | .59 |  |  |
| 39 | .74 |  |  |
| 40 | .54 |  |  |
| 41 |  | .70 |  |
| 42 | .43 |  |  |
| 43 | .48 |  |  |
| 44 | .53 |  |  |
| 45 |  | .78 |  |
| 46 |  | .70 |  |
| 47 | .54 |  |  |
| 48 |  |  | .56 |
| 49 | .52 |  |  |
| 50 | .65 |  |  |
| 51 | .66 |  |  |
| 52 |  | .83 |  |
| 53 | .64 |  |  |
| 54 | .65 |  |  |
| 55 |  | .58 |  |
| 56 | .65 |  |  |
| 57 | .41 |  |  |
| 58 | .75 |  |  |
| 59 | .48 |  |  |

*Note*. DSS = DBT Skills Subscale; DCS1 = Dysfunctional Coping Subscale 1 - general dysfunctional coping; DCS2 = Dysfunctional Coping Subscale 2 - blaming others.

Table B2

*Standardized Estimates of the Thresholds of the Non-Invariant DSS Items*

| Item | Threshold | BPD individuals | | |  | Non-clinical controls | | |
| --- | --- | --- | --- | --- | --- | --- | --- | --- |
|  |  | Estimate | SE | *p* |  | Estimate | SE | *p* |
| 6 | 1 | -1.416 | 0.129 | <.001 |  | -0.710 | 0.141 | <.001 |
|  | 2 | -0.391 | 0.090 | <.001 |  | -0.006 | 0.115 | .960 |
|  | 3 | 0.458 | 0.091 | <.001 |  | 1.208 | 0.145 | <.001 |
| 19 | 1 | -1.265 | 0.119 | <.001 |  | -1.648 | 0.223 | <.001 |
|  | 2 | -0.198 | 0.088 | .025 |  | -0.998 | 0.137 | <.001 |
|  | 3 | 1.115 | 0.111 | <.001 |  | 0.306 | 0.123 | .013 |
| 29 | 1 | 0.049 | 0.088 | .575 |  | 0.641 | 0.131 | <.001 |
|  | 2 | 0.599 | 0.094 | <.001 |  | 1.218 | 0.153 | <.001 |
|  | 3 | 1.212 | 0.116 | <.001 |  | 2.013 | 0.251 | <.001 |
| 42 | 1 | -0.086 | 0.088 | .327 |  | -0.688 | 0.136 | <.001 |
|  | 2 | 0.675 | 0.095 | <.001 |  | 0.152 | 0.121 | .209 |
|  | 3 | 1.565 | 0.140 | <.001 |  | 1.154 | 0.145 | <.001 |

*Note*. DSS = DBT Skills Subscale.

Table B3

*Standardized Estimates of the Thresholds of the Non-Invariant DCS Items*

| Item | Threshold | BPD individuals | | |  | Non-clinical controls | | |
| --- | --- | --- | --- | --- | --- | --- | --- | --- |
|  |  | Estimate | SE | *p* |  | Estimate | SE | *p* |
| 3 | 1 | -1.968 | 0.188 | <.001 |  | -1.993 | 0.170 | <.001 |
|  | 2 | -1.139 | 0.112 | <.001 |  | -0.810 | 0.133 | <.001 |
|  | 3 | 0.049 | 0.088 | .575 |  | 0.608 | 0.171 | <.001 |
| 7 | 1 | 0.098 | 0.088 | .263 |  | -0.442 | 0.142 | .002 |
|  | 2 | 0.754 | 0.097 | <.001 |  | 0.594 | 0.194 | .002 |
|  | 3 | 1.565 | 0.140 | <.001 |  | 1.416 | 0.312 | <.001 |
| 12 | 1 | -0.160 | 0.088 | .069 |  | -0.621 | 0.148 | <.001 |
|  | 2 | 0.417 | 0.091 | <.001 |  | 0.388 | 0.178 | .029 |
|  | 3 | 1.450 | 0.131 | <.001 |  | 1.533 | 0.400 | <.001 |
| 17 | 1 | -1.890 | 0.177 | <.001 |  | -1.492 | 0.149 | <.001 |
|  | 2 | -1.352 | 0.124 | <.001 |  | -0.680 | 0.149 | <.001 |
|  | 3 | -0.261 | 0.089 | .003 |  | 0.444 | 0.184 | .016 |
| 20 | 1 | -2.062 | 0.204 | <.001 |  | -1.874 | 0.161 | <.001 |
|  | 2 | -1.565 | 0.140 | <.001 |  | -0.804 | 0.138 | <.001 |
|  | 3 | -0.499 | 0.092 | <.001 |  | 0.305 | 0.160 | .057 |
| 37 | 1 | -1.322 | 0.122 | <.001 |  | -0.616 | 0.147 | <.001 |
|  | 2 | -0.929 | 0.103 | <.001 |  | 0.003 | 0.158 | .984 |
|  | 3 | 0.037 | 0.088 | .674 |  | 1.290 | 0.267 | <.001 |
| 41 | 1 | -1.705 | 0.154 | <.001 |  | -1.779 | 0.162 | <.001 |
|  | 2 | -1.007 | 0.106 | <.001 |  | -0.685 | 0.154 | <.001 |
|  | 3 | 0.351 | 0.090 | <.001 |  | 0.921 | 0.242 | <.001 |
| 48 | 1 | -0.236 | 0.089 | .008 |  | -0.986 | 0.137 | <.001 |
|  | 2 | 0.444 | 0.091 | <.001 |  | 0.061 | 0.145 | .672 |
|  | 3 | 1.450 | 0.131 | <.001 |  | 1.520 | 0.283 | <.001 |

*Note*. DCS = Dysfunctional Coping Subscale.

Table B4

*Item-rest Correlations* (*r*_ir_) *and Mean Scores of the DSS items*

| Item | Total group | | |  | | BPD | | |  | Non-clinical | |
| --- | --- | --- | --- | --- | --- | --- | --- | --- | --- | --- | --- |
|  | *r*_ir_ | *M* |  | | *r*_ir_ | | *M* |  | | *r*_ir_ | *M* |
| 1 | .42 | 1.48 |  | | .41 | | 1.45 |  | | .42 | 1.54 |
| 2 | .52 | 1.34 |  | | .59 | | 1.20 |  | | .35 | 1.61 |
| 4 | .58 | 1.75 |  | | .52 | | 1.64 |  | | .66 | 1.96 |
| 6 | .32 | 1.77 |  | | .33 | | 1.90 |  | | .45 | 1.51 |
| 9 | .54 | 1.65 |  | | .51 | | 1.57 |  | | .56 | 1.81 |
| 10 | .41 | 1.93 |  | | .36 | | 1.85 |  | | .46 | 2.10 |
| 11 | .40 | 1.58 |  | | .34 | | 1.46 |  | | .46 | 1.83 |
| 13 | .29 | 1.94 |  | | .24 | | 1.95 |  | | .41 | 1.93 |
| 16 | .57 | 1.82 |  | | .50 | | 1.77 |  | | .69 | 1.93 |
| 18 | .53 | 1.49 |  | | .49 | | 1.37 |  | | .58 | 1.71 |
| 19 | .63 | 1.85 |  | | .61 | | 1.61 |  | | .67 | 2.34 |
| 21 | .46 | 1.52 |  | | .40 | | 1.56 |  | | .61 | 1.43 |
| 22 | .49 | 1.38 |  | | .51 | | 1.47 |  | | .55 | 1.22 |
| 23 | .59 | 1.63 |  | | .58 | | 1.42 |  | | .59 | 2.03 |
| 26 | .44 | 1.86 |  | | .37 | | 1.88 |  | | .64 | 1.83 |
| 27 | .49 | 1.65 |  | | .48 | | 1.62 |  | | .51 | 1.72 |
| 29 | .23 | 0.73 |  | | .31 | | 0.87 |  | | .21 | 0.47 |
| 31 | .33 | 2.16 |  | | .24 | | 2.11 |  | | .49 | 2.25 |
| 33 | .43 | 1.35 |  | | .35 | | 1.29 |  | | .58 | 1.45 |
| 34 | .46 | 1.65 |  | | .40 | | 1.60 |  | | .57 | 1.76 |
| 35 | .31 | 2.04 |  | | .26 | | 2.10 |  | | .45 | 1.93 |
| 36 | .48 | 1.45 |  | | .48 | | 1.44 |  | | .52 | 1.46 |
| 38 | .51 | 1.57 |  | | .52 | | 1.53 |  | | .50 | 1.64 |
| 39 | .67 | 1.63 |  | | .66 | | 1.59 |  | | .69 | 1.71 |
| 40 | .49 | 1.46 |  | | .46 | | 1.39 |  | | .52 | 1.58 |
| 42 | .38 | 1.05 |  | | .32 | | 0.84 |  | | .41 | 1.45 |
| 43 | .43 | 1.61 |  | | .38 | | 1.50 |  | | .49 | 1.83 |
| 44 | .48 | 1.52 |  | | .48 | | 1.55 |  | | .52 | 1.47 |
| 47 | .49 | 1.90 |  | | .45 | | 1.83 |  | | .56 | 2.02 |
| 49 | .46 | 1.49 |  | | .38 | | 1.38 |  | | .58 | 1.71 |
| 50 | .57 | 1.35 |  | | .54 | | 1.20 |  | | .62 | 1.65 |
| 51 | .58 | 1.55 |  | | .59 | | 1.50 |  | | .58 | 1.65 |
| 53 | .55 | 1.29 |  | | .56 | | 1.13 |  | | .50 | 1.59 |
| 54 | .57 | 1.63 |  | | .57 | | 1.57 |  | | .56 | 1.74 |
| 56 | .57 | 1.62 |  | | .57 | | 1.56 |  | | .57 | 1.75 |
| 57 | .40 | 1.27 |  | | .30 | | 1.18 |  | | .55 | 1.46 |
| 58 | .68 | 1.58 |  | | .64 | | 1.48 |  | | .74 | 1.79 |
| 59 | .42 | 1.79 |  | | .39 | | 1.71 |  | | .48 | 1.94 |

*Note*. DSS = DBT Skills Subscale.

Table B5

*Item-rest Correlations* (*r*_ir_) *and Mean Scores of the DCS1 items*

| Item | Total group | | |  | | BPD | | |  | Non-clinical | |
| --- | --- | --- | --- | --- | --- | --- | --- | --- | --- | --- | --- |
|  | *r*_ir_ | *M* |  | | *r*_ir_ | | *M* |  | | *r*_ir_ | *M* |
| 3 | .59 | 2.05 |  | | .40 | | 2.33 |  | | .52 | 1.50 |
| 5 | .41 | 2.17 |  | | .36 | | 2.31 |  | | .32 | 1.89 |
| 8 | .49 | 1.52 |  | | .40 | | 1.77 |  | | .41 | 1.04 |
| 12 | .42 | 0.83 |  | | .38 | | 0.98 |  | | .42 | 0.55 |
| 14 | .49 | 1.97 |  | | .42 | | 2.17 |  | | .42 | 1.57 |
| 17 | .71 | 2.06 |  | | .48 | | 2.49 |  | | .63 | 1.21 |
| 20 | .71 | 2.20 |  | | .44 | | 2.61 |  | | .67 | 1.38 |
| 25 | .70 | 1.86 |  | | .56 | | 2.15 |  | | .71 | 1.27 |
| 32 | .44 | 1.79 |  | | .36 | | 1.97 |  | | .42 | 1.44 |
| 37 | .57 | 1.74 |  | | .27 | | 2.22 |  | | .45 | 0.81 |
| 41 | .61 | 1.87 |  | | .44 | | 2.16 |  | | .52 | 1.30 |
| 45 | .67 | 2.05 |  | | .54 | | 2.41 |  | | .52 | 1.33 |
| 46 | .64 | 1.79 |  | | .52 | | 2.08 |  | | .60 | 1.22 |
| 52 | .71 | 2.06 |  | | .59 | | 2.37 |  | | .65 | 1.46 |
| 55 | .54 | 1.69 |  | | .42 | | 1.92 |  | | .50 | 1.23 |

*Note*. DCS1 = Dysfunctional Coping Subscale 1 - general dysfunctional coping.

Table B6

*Item-rest Correlations* (*r*_ir_) *and Mean Scores of the DCS2 items*

| Item | Total group | | |  | | BPD | | |  | Non-clinical | |
| --- | --- | --- | --- | --- | --- | --- | --- | --- | --- | --- | --- |
|  | *r*_ir_ | *M* |  | | *r*_ir_ | | *M* |  | | *r*_ir_ | *M* |
| 7 | .51 | 0.68 |  | | .51 | | 0.75 |  | | .45 | 0.54 |
| 15 | .52 | 1.21 |  | | .53 | | 1.37 |  | | .41 | 0.90 |
| 24 | .63 | 1.46 |  | | .62 | | 1.58 |  | | .59 | 1.20 |
| 28 | .55 | 1.49 |  | | .52 | | 1.72 |  | | .52 | 1.04 |
| 30 | .68 | 0.98 |  | | .68 | | 1.11 |  | | .60 | 0.73 |
| 48 | .50 | 0.98 |  | | .51 | | 1.00 |  | | .55 | 0.96 |

*Note*. DCS2 = Dysfunctional Coping Subscale 2 - blaming others.
